# Predictive models for type 2 diabetes onset in middle-aged subjects with the metabolic syndrome

**DOI:** 10.1186/1758-5996-5-36

**Published:** 2013-07-15

**Authors:** Michal Ozery-Flato, Naama Parush, Tal El-Hay, Žydrūnė Visockienė, Ligita Ryliškytė, Jolita Badarienė, Svetlana Solovjova, Milda Kovaitė, Rokas Navickas, Aleksandras Laucevičius

**Affiliations:** 1 Machine Learning and Data Mining group, IBM Research - Haifa, Mount Carmel, Haifa 3498825, Israel; 2 Centre of Endocrinology, Vilnius University Hospital Santariškių Klinikos, Santariskiu g. 2, Vilnius LT-08661, Lithuania; 3 Centre of Cardiology and Angiology, Vilnius University Hospital Santariškių Klinikos, Santariskiu g. 2, Vilnius LT-08661, Lithuania; 4 Vilnius University, Medical Faculty, M. K. Ciurlionio g. 21, Vilnius LT-03101, Lithuania

**Keywords:** Metabolic syndrome, Type 2 diabetes mellitus, Risk assessment, Biomarkers, Arterial markers, Predictive models

## Abstract

**Objective:**

To investigate the predictive value of different biomarkers for the incidence of type 2 diabetes mellitus (T2DM) in subjects with metabolic syndrome.

**Methods:**

A prospective study of 525 non-diabetic, middle-aged Lithuanian men and women with metabolic syndrome but without overt atherosclerotic diseases during a follow-up period of two to four years. We used logistic regression to develop predictive models for incident cases and to investigate the association between various markers and the onset of T2DM.

**Results:**

Fasting plasma glucose (FPG), body mass index (BMI), and glycosylated haemoglobin can be used to predict diabetes onset with a high level of accuracy and each was shown to have a cumulative predictive value. The estimated area under the receiver-operating characteristic curve (AUC) for this combination was 0.92. The oral glucose tolerance test (OGTT) did not show cumulative predictive value. Additionally, progression to diabetes was associated with high values of aortic pulse-wave velocity (aPWV).

**Conclusion:**

T2DM onset in middle-aged metabolic syndrome subjects can be predicted with remarkable accuracy using the combination of FPG, BMI, and HbA_1c_, and is related to elevated aPWV measurements.

## Background

Metabolic syndrome (MetS) is a complex disorder defined by a cluster of interconnected factors that increase the risk of cardiovascular (CV) atherosclerotic diseases and type 2 diabetes mellitus (T2DM). The presence of MetS as a risk factor for T2DM has been examined in numerous population-based studies [1-5]. The meta-analysis of prospective studies shows MetS to be associated with an approximately five times higher risk for incident T2DM in many different populations, regardless of how the MetS is defined [6]. Impaired fasting glucose (IFG) and impaired glucose tolerance (IGT) are shown to be strong predictors of T2DM in many studies [7-9]. Other components of MetS, particularly waist circumference (WC), body mass index (BMI), and triglycerides were shown to be associated with incidence of T2DM in cohorts composed of subjects with high post-prandial glucose [10] and in the general population [11]. A recent study in patients with manifest atherosclerosis revealed that the presence of ≥ 3 metabolic risk factors or the presence of a high waist circumference alone are associated with increased risk for developing T2DM [12]. The combined presence of ≥ 3 metabolic risk factors and high waist circumference is associated with a 10-fold increased risk of future T2DM [12].

To date, only limited information is available on the predictors of T2DM in the group of patients that are already diagnosed with MetS but no overt atherosclerotic disease. While the majority of available studies report associations between the incidence of T2DM and the presence of MetS or other risk factors, the analysis of the predictive and cumulative value of these factors is lacking. The aim of our study was to investigate the predictive value of different clinical markers, including the ones described above, for T2DM onset in subjects with MetS before the manifestation of atherosclerotic disease.

## Methods

### Subject recruitment

All patients included in our study were recruited between 2007 and 2011 from the Lithuanian High Cardiovascular Risk (LitHiR) primary prevention programme [13]. This long-term programme has focused on employable-aged women (aged 50–65) and men (aged 40–55) without overt cardiovascular disease. Cardiovascular disease was defined as angina pectoris, known coronary stenosis, myocardial infarction**,** coronary artery bypass grafting, percutaneous coronary intervention, transient ischemic attack or stroke, and peripheral artery disease. As part of the programme, a two-level approach involving primary healthcare institutions (PHCI) and specialized cardiovascular prevention units (CVPU) was applied. Five secondary-level institutions having CVPU participated in the LitHiR programme across Lithuania, including the Vilnius University Hospital Santariškių Klinikos. Participants of the first level of the programme were recruited in three ways. The first group consisted of people registered in PHCI and invited by general practitioners to participate in the programme. The second group consisted of people who visited PHCIs for reasons other than cardiovascular problems. The third group included people who found out about the programme via local mass media. All participants had to match the programme criteria. After cardiovascular risk evaluation at the PHCI level, subjects for whom high cardiovascular risk was established were sent for additional examination and treatment plans in the CVPUs (secondary level). High cardiovascular risk was defined as having one or more of the following conditions: 1) a Systematic Coronary Risk Evaluation (SCORE) [14] risk assessment of over 11, 2) diabetes, 3) metabolic syndrome, 4) positive family history of cardiovascular disease and/or 4) severe dyslipidemia.

The number of PHCIs taking part in this program was 385/420, which comprise 91.6% of all PHCI in Lithuania. From 2006 to 2010, 266,391 patients were examined overall. Out of those patients, our cohort includes 2891 [1072 (37%) men and 1819 (63%) women] patients who were diagnosed with MetS and referred to the CVPU at the Vilnius University Hospital Santariškių Klinikos for additional assessment, risk stratification, and setting up of a prevention plan.

We carried out follow-up calls between January 2011 and August 2011 for 650 out of the 2891 subjects with MetS initially referred to the CPVU in Vilnius University Hospital Santariškių Klinikos. These follow-up calls were made with preference to subjects who were examined earlier in the programme. After we excluded four subjects whose follow-up periods were less than two years, the median of the follow-up period was 3.3 years. We also excluded 117 participants who already had diabetes at the baseline examination and 4 participants with missing information on their diabetic status. As a result, the final study cohort consisted of 525 individuals, with 187 (36%) men and 338 (64%) women.

The study was approved by the Local Ethics Committee of the Vilnius University Hospital Santariškių Klinikos.

### Diagnosis of MetS

We diagnosed patients as having MetS if they met three or more of the revised National Cholesterol Education Program Adult Treatment Panel III (NCEP ATPIII) criteria [15,16]:

– Waist circumference ≥ 102 cm in men, ≥ 88 cm in women

– Triglycerides ≥ 1.7 mmol/L

– High-density lipoprotein cholesterol < 1.03 mmol/L in men, < 1.29 mmol/L in women

– Blood pressure (BP) ≥ 130/85 mmHg

– Fasting plasma glucose (FPG) ≥ 5.6 mmol/L

We calculated the MetS score as the sum of MetS components present.

### Baseline examinations

All participants in our study underwent a baseline examination, which included gathering information on their medical history, physical examination, risk profile and lifestyle assessment, evaluation of cardiovascular (CV) family history, 12-lead electrocardiogram (ECG), laboratory blood tests, and non-invasive assessment of arterial markers of subclinical atherosclerosis. Weight, height, and waist circumference were measured with the subject wearing light clothing and without shoes. BMI was calculated as weight in kilograms divided by the square of height in meters. Blood pressure was measured after the patient rested at least five minutes, using an oscillometric semiautomatic device (Schiller Argus VCM) with a standard bladder (12–13 cm long and 35 cm wide), validated according to standardized mercury sphygmomanometer. We took at least one measurement on each arm and additional measurements if the first two were significantly different. The higher value was taken as the reference one and the average of the two highest values, if measured more than twice. Assessment of arterial stiffness was carried out by applanation tonometry (Sphygmocor v.7.01, AtCor Medical).

Information about smoking and drug use was collected by a questionnaire. Current smoking was recorded if the subject smoked at least one cigarette a day. Positive CV family history was recorded if first-degree relatives of the patient had any CV events at a young age (men ≤ 45 years, women ≤ 55 years old).

### Laboratory tests and assessment of glucose metabolism

Venous blood samples were collected after patients completed a 12-hour fast. Serum cholesterol [17,18], triglycerides [19,20], and plasma glucose concentrations were determined enzymatically. High-density lipoprotein cholesterol was analyzed by the Accelerator Selective Detergent method (Architect ci8200; Abbott Laboratories, Abbott Park, Illinois, USA). Low-density lipoprotein cholesterol was calculated with the Friedewald formula [21]. High-sensitivity serum C-reactive protein (hs-CRP) was analyzed by a latex turbidimetric immunoassay kit (Architect ci8200; Abbott Laboratories, Abbott Park, Illinois, USA). Multigent HbA_1c_ was determined by turbidimetric microparticle immunoinhibition assay (Architect ci8200; Abbott Laboratories, Abbott Park, Illinois, USA). Plasma fasting and oral glucose tolerance test (OGTT) insulin were measured by chemiluminescent microparticle immunoassay (CMIA) (Architect ci8200; Abbott Laboratories, Abbott Park, Illinois, USA). A standard 75-g OGTT was carried out after patients completed a 12-hour overnight fast. Plasma glucose and insulin concentrations were measured at 0 and 120 minutes. The examination protocol allowed the omission of OGTT, HbA_1c_, and fasting insulin tests for patients with FPG < 5.6.

We classified the subjects into various categories of glucose tolerance using the WHO criteria [22]. Normal glucose tolerance (NGT) was defined by fasting glucose <6.1 mmol/l and 2-h OGTT glucose <7.8 mmol/l. Impaired fasting glucose was defined by fasting glucose ≥ 6.1 mmol/l and <7.0 mmol/l and 2-h OGTT glucose <7.8 mmol/l. Impaired glucose tolerance was defined by fasting glucose <7.0 mmol/l and 2-h OGTT glucose between 7.8 and 11.0 mmol/l inclusive. Diabetes was defined by fasting glucose ≥7.0 mmol/l and/or 2-h OGTT glucose ≥11.1 mmol/l.

### Insulin resistance indices

In this study, we considered four surrogate indices for the assessment of insulin resistance (IR) or insulin sensitivity. The Homeostasis Model Assessment insulin resistance (HOMA-IR) index [23] was calculated as fasting insulin [μU/ml] × FPG [mmol/l] / 22.5. The quantitative insulin-sensitivity check index (QUICKI) index [24] was calculated as 1/[log(fasting insulin [μU/ml]) + log(FPG [mg/dl])]. The Cederholm insulin sensitivity index (ISI), which represents peripheral insulin sensitivity, was calculated as ISI_Cederholm_ = 75000 + (G_0_-G_120_) × 1.15 × 180 × 0.19 × weight/120 × G_mean_ × log (I_mean_) [25], where G_0_ and G_120_ are plasma glucose (mmol/l) concentrations at 0 and 120 minutes, and G_mean_ and I_mean_ are the mean glucose (mmol/l) and insulin (mU/l) values calculated from values at 0 and 120 minutes. Finally, the Matsuda insulin sensitivity index, which reflects a composite estimate of hepatic and muscle insulin sensitivity, was calculated as ISI_Matsuda_ = 10,000 / sqrt (G_0_ x I_0_ x G_120_ x I_120_) [26,27], where G_0_, G_120_, and I_0_, I_120_ are the plasma glucose (mg/dl) and the plasma insulin (μU/ml) concentrations respectively at time 0 and 120 minutes.

### Statistical analysis

We conducted descriptive statistics on the study cohort at the baseline; we calculated the mean and standard deviation (SD) for the continuous variables and the frequency and proportion for the categorical variables. The investigated set of variables included: age, gender, smoking status (never, former, current), BMI, waist circumference, weight, FPG, HbA_1c_, fasting plasma insulin, OGTT glucose, OGTT insulin, serum triglycerides, total cholesterol, HDL cholesterol, LDL cholesterol, lipid treatment (1=yes, 0=no), hs-CRP, aortic and radial pulse wave velocity (aPWV, rPWV), aortic augmentation index adjusted for heart rate 75 beats per minute (AIx@75), mean arterial pressure (MAP), MetS score, HOMA-IR, QUICKI, ISI_Matsuda_, and ISI_Cederholm_.

We measured the association between each variable and the development of T2DM by calculating gender-adjusted odds ratios (ORs). We initially included the gender variable in any set of predictors tested. We investigated the dependency between variables and their cumulative contribution to the prediction based on their combined logistic regression model. P values are based on two-sided tests with a cutoff for statistical significance of 0.05. To address the inherent problem of multiple hypotheses testing, we applied the Bonferroni correction, multiplying the P value by the number of independent tests.

We performed all tests on complete data; that is, excluding those patients with data missing for the relevant variables. We used Little’s [28] missing completely at random (MCAR) test to identify systematic differences between the missing values and the observed values. A significant P value in Little’s MCAR test, indicating the existence of such systematic differences, means that it is plausible that data are missing at random (MAR), but not completely at random (MCAR). In these cases, since restricting analyses to complete cases can introduce bias, we validated the results using multiple imputation [29,30]. We used the fully conditional specification [31] imputation method, as implemented in SPSS MULTIPLE IMPUTATION command, to make 20 complete datasets. We then combined (pooled) multiple analyses’ results using Rubin’s Rules [30,32].

In a separate analysis, we considered the tested variables using a stepwise algorithm that automatically selected variables for a multivariate logistic regression model. This method used the Bayesian Information Criterion (BIC), which assesses model fit based on a log-likelihood function [33]. The model with the lowest value of BIC is the one preferred. We took a “forward” approach, starting with a model initialized with the gender variable, adding at each step one variable that maximally reduced the BIC statistic and terminated when the BIC statistic stopped decreasing. We estimated the accuracy of the predictive models using leave-one-out cross-validation; that is, each subject in its turn was used as a validation set, while the remaining subjects were used to generate the model. We assessed the predictive discrimination of the model using the receiver-operating characteristic (ROC) curve of the scores of all subjects by plotting the sensitivity against the corresponding false positive rate. We used the area under the ROC curve, calculated by the trapezoidal rule, to measure how well a model predicts the development of T2DM. The model generation involved a preliminary step of data imputation for missing values using mean values. We also used an alternative analyses using K-nearest-neighbors data imputation, which yielded similar results; only the mean imputation results are presented.

All statistical and modeling analysis was done using MATLAB 7.13 (R2011b) and SPSS Statistics 19.0.0.

## Results

We observed data from 187 men and 338 women with mean ±SD ages at a baseline of 48±4 and 57±4 years for an average of 3.2 and 3.3 years, respectively. During the follow-up period of 2 to 4 years, a total of 32 subjects progressed to diabetes: 16 (8.5%) of the 187 men and 16 (5%) of the 338 women. Table 1 shows the baseline characteristics of the two groups: progressors and nonprogressors.

**Table 1:** Baseline characteristics

**Variable**	**Complete case**	**Men**		**Women**	
		**Non-progressors**	**Progressors**	**Non-progressors**	**Progressors**
FPG (mmol/L)	521 (99%)	5.9 (0.8)	7.1 (1.2)	5.7 (0.6)	6.7 (0.6)
BMI (kg/m^2^)	524 (100%)	30.3 (3.8)	35.6 (5.4)	30.7 (4.6)	34.6 (5.4)
Waist circumference (cm)	520 (99%)	106.6 (9.4)	116.5 (9.7)	100.8 (9.6)	108.9 (7.3)
OGTT glucose (mmol/L)	425 (81%)	5.4 (1.6)	6.9 (1.8)	6.3 (1.7)	7.8 (1.8)
HbA_1c_ (%)	405 (77%)	5.6 (0.2)	6.0 (0.5)	5.7 (0.3)	5.9 (0.2)
Quicki	326 (62%)	0.1 (0.0)	0.1 (0.0)	0.1 (0.0)	0.1 (0.0)
MetS score (0–5)	525 (100%)	3.3 (1.0)	3.8 (0.8)	3.4 (1.0)	4.3 (0.7)
Weight (kg)	524 (100%)	95.4 (13.9)	107.6 (16.2)	80.0 (12.9)	87.6 (12.9)
ISI_Matsuda_	299 (57%)	7.8 (5.4)	3.8 (2.4)	6.6 (4.8)	3.3 (2.2)
OGTT insuline (pmol/l)	301 (57%)	254.5 (199.3)	473.7 (209.2)	415.5 (345.8)	694.7 (492.8)
HOMA-IR	326 (62%)	3.2 (1.8)	5.7 (2.3)	3.4 (2.6)	4.9 (2.0)
Fasting insuline (pmol/l)	326 (62%)	84.3 (43.0)	131.9 (49.3)	88.0 (61.6)	115.5 (49.0)
HDL cholesterol (mmol//l)	523 (100%)	1.2 (0.3)	1.1 (0.2)	1.4 (0.3)	1.2 (0.2)
LDL cholesterol (mmol/L)	524 (100%)	4.3 (1.2)	3.7 (1.1)	4.8 (1.3)	4.4 (0.9)
Total cholesterol (mmol/l)	525 (100%)	6.7 (1.4)	6.1 (1.3)	7.1 (1.4)	6.7 (1.2)
hs-CRP (mg/L)	502 (96%)	4.0 (6.8)	3.9 (4.3)	2.9 (3.2)	7.2 (13.7)
ISI_Cederholm_	299 (57%)	75088.2 (1048.6)	75363.3 (1453.0)	74441.7 (1223.9)	73737.3 (1848.3)
Age (years)	525 (100%)	48.0 (4.0)	49.1 (4.4)	56.9 (4.1)	56.9 (3.6)
Smoking status	522 (99%)				
Never		82 (49%)	8 (50%)	267 (83%)	15 (94%)
Former		13 (8%)	0 (0%)	7 (2%)	0 (0%)
Current		73 (43%)	8 (50%)	48 (15%)	1 (6%)
Triglycerides (mmol/l)	525 (100%)	2.7 (2.4)	3.0 (1.6)	2.1 (2.2)	2.2 (0.9)
Statin treatment	501 (95%)	163 (98%) 4 (2%)	13 (100%) 0 (0%)	303 (99%) 3 (1%)	15 (100%) 0 (0%)
aPWV (m/s)	480 (91%)	8.6 (1.4)	9.3 (1.8)	8.8 (1.4)	9.1 (1.6)
rPWV	496 (94%)	9.1 (1.2)	9.0 (1.5)	8.9 (1.3)	8.5 (1.3)
MAP (mmHg)	493 (94%)	107.5 (13.2)	103.9 (9.9)	106.7 (14.6)	106.4 (16.3)
Aix@75 (%)	499 (95%)	18.1 (8.8)	16.3 (9.1)	30.3 (7.8)	28.1 (14.1)

### Missing values

One hundred (19%) of the subjects had missing OGTT glucose test values, and 120 (23%) of the subjects had missing HbA_1c_ values. Applying Little’s MCAR test on the entire set of variables had a significant result (χ^2^(636)=589.6, P<0.001). Repeating Little’s MCAR test after the exclusion of these variables led to non-significant results (χ^2^(180)=179.6, P>0.1). These results were expected as the examination protocol recommended OGTT, HbA_1c_ and fasting insulin tests in patients with higher FPG values. Since missing values were not missing completely at random (MCAR), we validated our results in multiple imputation analysis (see “Statistical Analysis” Section).

### Baseline classification of subjects

At the baseline, 237 (45%) had NGT, 99 (19%) had impaired fasting glucose (IFG), and 67 (13%) had impaired glucose tolerance (IGT). Twenty two (4%) subjects had FPG ≥7 mmol/l, but were diagnosed as non-diabetic by an endocrinologist, based on additional test results (including former fasting glucose test). One hundred (19%) of the subjects were not classified mainly due to missing OGTT glucose test values. In the multiple imputation analysis, most of the unclassified patients were in the NGT group, which then increased to 60% (95% confidence interval [CI] 58-61%) of the patients. The IFG and the IGT group contained 20% (CI 20-21%) and 16% (CI 14.5-17 %) of the patients, respectively.

### Impaired fasting glucose and impaired glucose tolerance

In this section, we report the results of multiple imputation analysis. Complete data analysis had similar results (not shown). The association of T2DM onset with the IFG and IGT groups was significant: the odds-ratio in the IFG group was 3.7 (CI 1.5-9.4 P= 0.006) and in the IGT group was 3.3 (CI 1.2-8.7, P=0.01). The odds ratios for T2DM onset were higher when the underlying criteria for FPG [≥6.1, <7 mmol/l] and OGTT glucose [≥6.1, <11 mmol/l] were combined: 11 (CI 3.2-38, P = 0.0001) in subjects satisfying at least one criterion, and 7.9 (CI = 2.8-22.4, P = 0.0001) in subjects satisfying both. The FPG criterion alone showed an even stronger association with T2DM onset: odds-ratio 12.3 (CI 4.1-37.4, P<0.0001).

### Identifying an effective set of predictors for T2DM

We found a combination of variables that effectively predicts T2DM using the following iterative analysis. Iteratively, after adjusting for previously included variables, we added to the set of predictors the strongest predictor for T2DM whose cumulative effect was shown to be significant (Bonferonni corrected P < 0.05, odds-ratio test). The iteration ended when no variable could be added. We report the results of multiple imputation analysis. Complete data analysis yielded similar results (not shown). Table 2 presents the odds-ratio results for all variables after they were adjusted for gender. In the first iteration, we identified 11 significant predictors (presented in decreasing order of their association): FPG, BMI, Waist circumference, OGTT glucose, HbA_1c_, Quicki, MetS score, Weight, IS_IMatsuda_, OGTT insuline, HOMA-IR, and Fasting Insuline. In the second iteration, after adjusting for FPG and gender, the BMI showed the most significant association. After the selection of BMI (third iteration), only HbA_1c_ remained a significant predictor. The final set included: gender, FPG, BMI and HbA_1c_. The selected variables: FPG, BMI and HbA_1c_, each showed a significant cumulative effect in the final model (FPG: P=0.000001; BMI: 0.00001; HbA_1c_: P=0.0004).

**Table 2:** ORs of the various investigated markers, adjusted for gender

**Variables**	**OR (95% CI) Bonferroni corrected P-value**		
	**All**	**Men**	**Women**
FPG	4.3 (2.6 - 7.2) p<0.0001	3.9 (1.9 - 8.1) p=0.01	4.8 (2.4 - 9.5) p=0.0002
BMI	1.2 (1.1 - 1.3) p<0.0001	1.3 (1.2 - 1.5) p=0.0005	1.2 (1.1 - 1.3) p=0.04
Waist circumference	1.09 (1.05 - 1.13) p<0.0001	1.1 (1.0 - 1.2) p=0.01	1.1 (1.0 - 1.1) p=0.1
OGTT glucose	1.6 (1.3 - 2.0) p=0.0002	1.6 (1.2 - 2.2) p=0.05	1.7 (1.2 - 2.3) p=0.04
HbA_1c_	13.0 (4.1 - 41.7) p=0.0003	33.1 (4.5 - 240.9) p=0.01	6.5 (1.5 - 27.6) NS
Quicki	0.00 (0.00 - 0.00) p=0.001	0.00 (0.00 - 0.00) p=0.1	0.00 (0.00 - 0.00) NS
MetS score	2.5 (1.6 - 3.8) p=0.001	1.8 (1.0 - 3.3) NS	3.4 (1.8 - 6.7) p=0.01
Weight	1.0 (1.0 - 1.1) p=0.003	1.1 (1.0 - 1.1) p=0.1	1.0 (1.0 - 1.1) NS
ISI_Matsuda_	0.6 (0.5 - 0.8) p=0.01	0.6 (0.4 - 0.9) NS	0.6 (0.4 - 0.9) NS
OGTT insuline	1.002 (1.001 - 1.003) p=0.02	1.00 (1.00 - 1.01)NS	1.002 (1.000 - 1.003) NS
HOMA-IR	1.3 (1.1 - 1.5) p=0.02	1.5 (1.1 - 2.0) NS	1.2 (1.0 - 1.3) NS
Fasting insuline	1.01 (1.00 - 1.01) NS	1.02 (1.00 - 1.03) NS	1.00 (1.00 - 1.01) NS
HDL_Ch	0.1 (0.0 - 0.6) NS	0.2 (0.0 - 2.5) NS	0.1 (0.0 - 0.7) NS
LDL cholesterol	0.7 (0.5 - 1.0) NS	0.6 (0.4 - 1.0) NS	0.8 (0.5 - 1.2) NS
Total cholesterol	0.7 (0.6 - 1.0) NS	0.7 (0.5 - 1.1) NS	0.8 (0.5 - 1.1) NS
hs-CRP	1.0 (1.0 - 1.1) NS	1.0 (0.9 - 1.1) NS	1.1 (1.0 - 1.2) NS
ISI_Cederholm_	0.99 (0.99 - 1.00) NS	1.00 (1.00 - 1.00) NS	1.00 (0.99 - 1.00) NS
Age	1.0 (0.9 - 1.1) NS	1.1 (0.9 - 1.2) NS	1.0 (0.9 - 1.1) NS
Smoking (never, former, current)	0.9 (0.6 - 1.4) NS	1.1 (0.6 - 1.8) NS	0.6 (0.2 - 1.7) NS
Triglycerides	1.0 (0.9 - 1.2) NS	1.0 (0.9 - 1.3) NS	1.0 (0.8 - 1.2) NS
Statin treatment (no, yes)	0.0 --	0.0 --	0.0 --

P-values are Bonferonni corrected. P>0.1 is marked insignificant (NS). For the binary statin treatment variable all incident cases had a negative value. Hence odds-ratio for statin treatment is 0, and the confidence interval and p-value are not defined.

### Model selection and accuracy estimation

**Figure 1:**
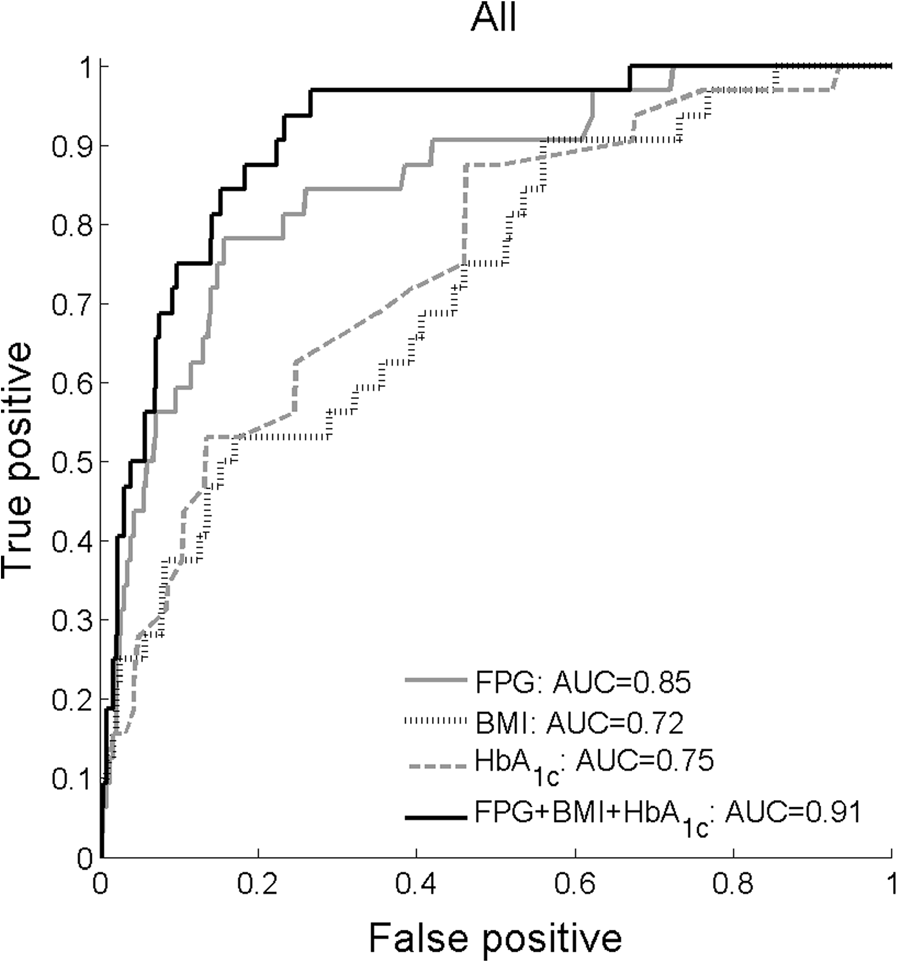
**Comparison of prediction models.** ROC curves of four diabetes onset prediction models: FPG-model, BMI-model, HbA_1c_-model, and a FPG-BMI-HbA_1c_-model. All models were adjusted for the gender variable.

In a separate analysis, we tested a model selection algorithm for building a predictive model for T2DM. This algorithm used a stepwise multivariate logistic regression with the Bayesian Information Criterion (BIC) measurement as a goodness-of-fit. To account for gender differences, the initial model contained the gender variable. Notably, the FPG-BMI-HbA_1c_ combination was consistently selected for all training sets. The overall estimated accuracy of the model was remarkably high (AUC=0.91). Figure 1 exemplifies the predictive power of FPG, BMI, and HbA_1c_, as well as the improvement in the prediction for their combined, gender-adjusted score, by plotting the ROC curves of the corresponding models.

### Comparison of BMI, waist circumference and weight

We tested the cumulative value of the obesity measures: BMI, waist circumference (WC), and weight, with respect to one another by combining them all into one model and adjusting for gender and FPG. Complete cases and multiple imputation analyses had similar results; only the latter is reported. Under this model, BMI had the most significant cumulative effect (P = 0.003, odds ratio test), compared to weight (P=0.03, odds ratio test), and waist (P>0.1, odds ratio test).

Additionally, we compared the estimated accuracy of three prediction models, each corresponding to one of the three of obesity measures, together with gender, FPG, and HbA_1c_. All three models had high estimated accuracy (AUC: BMI: 0.91, Weight=0.9, Waist=0.92). In summary, although BMI showed the strongest cumulative effect, all three obesity measures exhibited comparable discrimination under a model that contains FPG, HbA_1c_, and gender.

### OGTT glucose and FPG

We compared the cumulative values of OGTT glucose and FPG with respect to each other by testing their combination. Complete cases and multiple imputation analysis were in agreement; we report results for the latter. We tested the cumulative effect of FPG and OGTT glucose in two settings: after adjustment to gender and after adjustment to gender, BMI, and HbA_1c_. In both cases, FPG exhibited a very significant cumulative effect (P< 0.00001, odds-ratio test). On the other hand, OGTT glucose showed a milder cumulative effect in the gender-adjusted model (P=0.007, odds-ratio test), and no significant effect when BMI and HbA_1c_ were added to the model (P> 0.1, odds-ratio test).

In an ROC analysis with cross validation, the FPG model exhibited better performance than the OGTT glucose model (AUC: FPG=0.83, OGTT glucose = 0.71). The combined model FPG-OGTT glucose did not show any improvement (AUC=0.83). This confirmed that in our cohort, FPG is superior to OGTT glucose in predicting T2DM, and that OGTT glucose shows no cumulative effect in a model that contains FPG.

### Association of diabetes with arterial markers of cardiovascular risk

We used the following measures of arterial stiffness as surrogate markers for cardiovascular risk (CVR) at baseline examination: aortic and radial pulse wave velocity (aPWV, rPWV) adjusted to the mean arterial pressure (MAP), and aortic augmentation index adjusted for a heart rate of 75 beats per minute (AIx@75). Complete case analysis showed that Aix@75 and rPWV markers have no significant association with either progression to diabetes or IGT/IFG pre-diabetes conditions. On the other hand, high aPWV values were significantly associated with the IGT condition at baseline (P=0.01; odds ratio test) and with progression to diabetes (P = 0.04; odds ratio test). Repeating the tests with multiple imputations yielded no significant results. As aPWV seemed to be missing completely at random (Little’s MCAR test), we repeated the multiple imputation analysis after restricting the data to patients with non-missing aPWV values, retaining 480 (91%) of the patients. This time, the results of the multiple imputation analysis matched the complete case analysis. Testing the association between aPWV and CRP yielded no significant result (P>0.1, Pearson correlation test, complete case, and multiple imputation analyses).

## Discussion

In this study, the combination of FPG, BMI, and HbA_1c_ was shown to be a powerful predictor for the development of T2DM in subjects with MetS. FPG was shown to be superior to OGTT glucose in predicting T2DM, with OGTT glucose showing no cumulative value to FPG. Our study is aligned with general population studies showing that both IGT and IFG are similarly associated with an increased risk of diabetes, and that risks are higher when IGT and IFG coexist [34]. IFG was more prevalent than IGT in our cohort, while the opposite trend is usually observed in the general population [34]. The higher rate of IFG can be attributed to the fact that our study cohort consisted of subjects with high metabolic risk, in whom higher values of FPG are expected. Our findings of FPG being a stronger predictor than OGTT glucose and that OGTT exhibited no cumulative value to FPG, are different from the reports of other studies [35,36]. This increased predication power of FPG can be explained by the high prevalence of elevated FPG in our group. Similar to [37-39], which studied long-term prediction of T2DM risk in the general population, our results do not support the need for performing a 2-h OGTT to pinpoint the possible candidates for future diabetes in MetS subjects.

BMI and HbA_1c_ were evaluated as predictors of diabetes in numerous studies. BMI is known to be a major predictor for T2DM in the general population [35], as well as in the MetS population [11]. The T2DM risk was shown to increase exponentially with HbA_1c_ in both genders [40]. In another large study, the model including both FPG and HbA_1c_ was shown to be more effective for T2DM prediction than models including FPG alone or HbA_1c_ alone [41]. Recently, a study confirmed that HbA_1c_ of ≥5.6% had an increased risk for progression to T2DM, independent of other confounding factors [42]. This supports our finding on the cumulative effect of HbA_1c_, with respect to FPG and HbA_1c_.

Our investigation of four common insulin resistance/sensitivity indices yielded that these are less predictive for T2DM than FPG and OGTT glucose, as previously indicated by other studies whose cohorts were characterized by a high rate of IFG [37]. The association of these indices with progression to T2DM became insignificant after adjusting for FPG. This is similar to another report [43], which tested the association of the HOMA-IR index with T2DM after adjustment for BMI and familial history.

Our applanation tonometry results correspond with previous studies concerning the association between aPWV and diabetes, and the lack of association between elevated augmentation index and the presence of diabetes [44]. Similar to previous reports [45], our study demonstrated that the association between increased aortic stiffness and glucose metabolism abnormalities (IGT) is already found in pre-diabetic stages, and that IGT is more strongly associated with cardiovascular risk than IFG. The increased aPWV in our study cannot be explained by the elevation of CRP, and is predominantly associated with elevated 2h-OGTT glucose measurements.

To the best of our knowledge, no previous study established a predictive model for a new onset of diabetes in subjects with MetS. Since we focused on middle-aged metabolic-syndrome subjects, a possible limitation of our study is that its results cannot be generalized to subjects without MetS. Our study was also limited by the size of our dataset (525 subjects) and by the short duration of the follow-up period (2 to 4 years), resulting in only 32 participants that developed diabetes during the follow-up period. The subsequent unbalanced ratio between progressors and non-progressors, together with the relatively small size of the dataset, led to higher uncertainty in assessing the level of the risk estimate for considered variables. Another drawback of our study is the lack of information on diabetes familial history, which was shown to be a strong predictor for T2DM in the general population as well as in MetS subjects [11]. As 2h-OGTT glucose was found to be inferior to FPG in predicting T2DM in MetS subjects, future studies should also consider 1h-OGTT glucose, which was found to be a stronger predictor than 2h-OGTT glucose in several studies [37,46].

## Conclusions

The main finding of our study suggests that simple measures, such as BMI, FPG, and HbA_1c_ can accurately predict the development of T2DM in subjects with MetS. Meta-analysis of data from many population-based studies has shown that MetS, regardless of how it is defined, is a significant predictor of incident diabetes in many different populations [6]. Our study added to the current knowledge that for subjects who already have MetS, no sophisticated tests are needed to accurately identify the risk of incident diabetes: fasting plasma glucose is the strongest predictor with BMI and glycosylated haemoglobin having cumulative value.

## Competing interests

The authors declare that they have no competing interests.

## Authors' contributions

MO researched data, wrote the manuscript, and is the guarantor of this work. NP researched data and wrote the manuscript. TE researched the data and reviewed/edited the manuscript. ZV wrote the manuscript. LR collected data and wrote the manuscript. JB, SS, and MK collected data and reviewed the manuscript. RN and AL reviewed/edited the manuscript. All authors read and approved the final manuscript.
